# Machine Learning Analysis of Handgun Transactions to Predict Firearm Suicide Risk

**DOI:** 10.1001/jamanetworkopen.2022.21041

**Published:** 2022-07-11

**Authors:** Hannah S. Laqueur, Colette Smirniotis, Christopher McCort, Garen J. Wintemute

**Affiliations:** 1 Violence Prevention Research Program, Department of Emergency Medicine, University of California, Davis, Sacramento; 2 California Firearm Violence Research Center, Sacramento

## Abstract

**Importance:**

Evidence suggests that limiting access to firearms among individuals at high risk of suicide can be an effective means of suicide prevention, yet accurately identifying those at risk to intervene remains a key challenge. Firearm purchasing records may offer a large-scale and objective data source for the development of tools to predict firearm suicide risk.

**Objective:**

To test whether a statewide database of handgun transaction records, coupled with machine learning techniques, can be used to forecast firearm suicide risk.

**Design, Setting, and Participants:**

This prognostic study used the California database of 4 976 391 handgun transaction records from 1 951 006 individuals from January 1, 1996, to October 6, 2015. Transaction-level random forest classification was implemented to predict firearm suicide risk, and the relative predictive power of features in the algorithm was estimated via permutation importance. Analyses were performed from December 1, 2020, to May 19, 2022.

**Main Outcomes and Measures:**

The main outcome was firearm suicide within 1 year of a firearm transaction, derived from California death records (1996-2016). With the use of California’s Dealer’s Records of Sale (1996-2015), 41 handgun, transaction, purchaser, and community-level predictor variables were generated.

**Results:**

There are a total of 4 976 391 transactions in the California’s Dealer’s Record of Sale database representing 1 951 006 individuals (1 525 754 men [78.2% of individuals]; mean [SD] age, 43.4 [13.9] years). Firearm suicide within 1 year occurred in 0.07% of handgun transactions (3278 transactions among 2614 individuals). A total of 38.6% of observed firearm suicides were among transactions classified in the highest-risk ventile (379 of 983 transactions), with 95% specificity. Among the small number of transactions with a random forest score above 0.95, more than two-thirds (24 of 35 [68.6%]) were associated with a purchaser who died by firearm suicide within 1 year. Important features included known risk factors, such as older age at first purchase, and previously unreported predictors, including distance to firearms dealer and month of purchase.

**Conclusions and Relevance:**

This prognostic study presented the first large-scale machine learning analysis of individual-level handgun transaction records. The results suggested the potential utility of such records in identifying high-risk individuals to aid suicide prevention efforts. It also identified handgun, individual, and community characteristics that have strong predictive relationships with firearm suicide and may warrant further study.

## Key Points

**Question:**

Can handgun purchasing records, coupled with machine learning techniques, be used to forecast firearm suicide risk?

**Findings:**

In this prognostic study of nearly 2 million individuals with handgun transaction records, among transactions classified in the riskiest 5%, close to 40% were associated with a purchaser who died by firearm suicide within 1 year. Among the small number of transactions with a random forest score of 0.95 and above, more than two-thirds were affiliated with a purchaser who died by firearm suicide within 1 year (24 of 35).

**Meaning:**

This study suggests that passively collected administrative data on handgun transactions may be used to inform targeted interventions based on risk stratification.

## Teaser

This prognostic study tests whether a statewide database of nearly 5 million handgun transaction records, coupled with machine learning techniques, can be used to forecast firearm suicide risk.

## Introduction

In 2020, 47 979 Americans died by suicide, 24 292 by firearm suicide.^1^ Firearms are by far the most lethal method of suicide,^2^ accounting for only 5% of suicidal acts, but more than half of suicide deaths.^3^ Approximately 9 of 10 suicide attempts with a firearm are fatal.^3^ Given this lethality, and the often impulsive nature of suicide attempts, firearm access has been identified as a major risk factor for suicide^4^ and point of intervention for suicide prevention.^5^

Research has established a clear and strong association between firearm acquisition and ownership and firearm suicide risk.^6,7^ A large-scale study of 26 million California residents followed up for more than 12 years found that men who owned handguns were 8 times more likely than men who did not own handguns to die by firearm suicide; women handgun owners were more than 35 times more likely than women who did not own handguns to die by firearm suicide.^6^ In the first 30 days after handgun acquisition, the rate of suicide among handgun owners was 100 times higher compared with nonowners, suggesting that acquisition itself is associated with elevated suicide risk.

Although limiting access to firearms among individuals at increased risk for suicide presents a critical opportunity to save lives, accurately identifying those at risk remains a key challenge. A meta-analysis of 50 years of research found that accuracy of predicting suicide attempts was only slightly better than chance.^8^ In recent years, however, with the increasing availability of large-scale data sets and advances in computational power, there have been new efforts to leverage machine learning techniques to improve predictions.^9^ Recent studies using electronic health records,^10,11,12^ veterans’ health data,^13,14^ and social media postings^15^ have shown promise, with machine learning approaches generally producing more accurate and informative predictions than clinical evaluations or traditional regression techniques.^16,17^

Firearm transaction records are a large-scale, objective, and potentially critical administrative data source that might be used to help identify individuals at risk of firearm suicide. To our knowledge, the present study is the first to use such data to develop a machine learning prediction algorithm. Specifically, we use random forest (RF) classification to test whether California’s Dealer’s Record of Sale (DROS) database of nearly 5 million handgun transaction records for 1 951 006 individuals can be used to identify those at elevated risk of firearm suicide and to examine what features are important in determining these predictions.

## Methods

A full description of the data and analytic approach are provided in the eMethods in the Supplement. This study followed the Transparent Reporting of a Multivariable Prediction Model for Individual Prognosis or Diagnosis (TRIPOD) reporting guideline for prediction modeling^18^ and was approved by the University of California, Davis institutional review board. Informed consent requirements were waived because this was a secondary data analysis of largely deidentified data posing minimal risk.

### Data Source

Our primary predictive variables were generated from DROS transaction records for handgun transfers from January 1, 1996, to October 6, 2015. The DROS records are maintained in the California Department of Justice Automated Firearms System database. Under California law, essentially all transfers of firearms must be done through a licensed firearms retailer, including transfers between private parties, sales at gun shows, gifts, loans, and the redemption of pawned or consigned weapons. The legal age to purchase a handgun in California is 21 years.^19^ Detailed records for handgun transactions have been recorded since 1996 and record-keeping for long guns began in 2014; thus, we restricted our analyses to handguns. The DROS records include purchaser identifiers, address, date and time of the transaction, identifiers for the seller, and the firearm caliber, type, make, and model.

### Outcome Measure

We identified firearm suicide deaths (*International Classification of Diseases, Ninth Revision* and *International Statistical Classification of Diseases and Related Health Problems, Tenth Revision* codes X72-X74) by linking purchasers in DROS to the California Department of Public Health’s Death Statistical Master File (1996-2016), using probabilistic matching on name, date of birth, and gender/sex. We forecast firearm suicide within 1 year of the transaction.

### Predictor Variables

We generated 41 predictor variables from the DROS transaction data. Several features related to the handgun, including handgun category (revolver vs semiautomatic pistol vs unknown); caliber, categorized into small (eg, .22, .25, or .32), medium (eg, .38 or 9 mm), and large (eg, .40, .44, or .45); and an indicator for “inexpensive” handgun, proxied by the manufacturer, selecting the bottom quartile of median prices found in the 2017 *Blue Book of Gun Values*.^20^

Transaction characteristics included a binary indicator for whether the handgun was purchased at a gun show (yes, no, or unknown), the transaction type (eg, sale, voluntary registration, pawn redemption, or law enforcement acquisition), and transaction status (sale approved, denied, or cancelled). We also calculated the number of purchases an individual had made in the prior 1, 2, 5, and 10 years. We included all individual purchaser demographic variables available in DROS: gender, race and ethnicity (American Indian, Asian, Asian Indian, Black, Cambodian, Chinese, Filipino, Guamanian, Hawaiian, Hispanic, Japanese, Korean, Laotian, Pacific Islander, Samoan, Vietnamese, White, other, and unknown), place of birth (California vs elsewhere), and age at the time of the transaction.

We geocoded the purchaser and dealer addresses to identify the associated census tracts and obtain community characteristics. We included several county-level characteristics associated with the purchaser’s address and dealer’s location. Using the California Death Statistical Masterfile, we generated a county-level ratio of firearm suicides divided by total suicides (a common proxy for firearm prevalence).^21^ We also included the mean suicide rate in the county during the 12 months prior to the month of the transaction and a moving monthly mean of the number of handguns purchased (as reported in DROS) in the county during the previous 12 months to estimate local firearm purchasing trends. We calculated the distance between the purchaser’s address and the location of dealer. Last, for both the purchaser and dealer address, we included rural-urban status based on the US Department of Agriculture’s Rural-Urban Continuum Codes. Analyses were performed from December 1, 2020, to May 19, 2022.

### Statistical Analysis

#### Algorithm Development

##### Random Forest

We implemented RF classification^22^ on the transaction-level data to predict firearm suicide within 1 year. Random forest has been shown to be among the strongest-performing classifiers^23^ and to frequently perform well on imbalanced data.^24^ It has been successfully applied in a range of contexts, including criminal offending,^25,26^ disease forecasting,^27^ and predicting suicide ideation.^15^

In brief, RF works by aggregating many hundreds of classification trees, each of which represents a recursive partitioning of the training data. Each tree is built from a bootstrapped sample of the training data. The tree creates binary splits based on a sample of predictor variables, drawn randomly at each partition, and selecting the best (ie, purest) split. The tree is grown until either purity or a node size of 1 is reached. Finally, the trees are aggregated to create the RF and each observation receives a predicted probability or score based on the proportion of trees that assign it to the positive class. Depending on the task, these scores are converted to a class label determined by the decision threshold. The default threshold is majority rule.^28^

##### Dealing With Imbalanced Data

The major challenge with predicting firearm suicide is the rarity of the event, resulting in extreme class imbalance. Undersampling is a well-established technique for dealing with imbalanced data, shown to improve an algorithm’s ability to isolate the event signal.^29,30,31^ This approach balances the data by decreasing the size of the majority class. We implemented undersampling within the RF algorithm by using a bootstrapped sample of the same size from each class (or strata) to create a balanced data set to grow each tree.

##### Algorithm Tuning

We tuned 2 RF parameters on the training set: the number of predictor variables to randomly select at each binary split and the number of trees in the forest, by maximizing the area under the receiver operator characteristic curve (AUC) within the caret package in R,^32^ version 6.0-86 (R Group for Statistical Computing).^33^

##### Algorithm Evaluation

We evaluated the algorithm with test-set AUC, area under the precision-recall curve (AUC-PR), sensitivity, specificity, and metrics that combine sensitivity and specificity that are commonly used on imbalanced classification problems^34^: F-score^35^ and Youden index.^36^ We also present the positive predictive value and negative predictive value.

#### Variable Importance

To shed some light on the “black box” algorithm, we present variable importance measures, which provide information on predictors’ contributions to classification accuracy. We focus on mean decrease in accuracy. This approach permutes each variable and calculates the difference in accuracy before and after permuting (averaged over all trees and normalized by the SD of the difference). In addition to the overall variable importance, we also present minority class–specific importance measures, which can be useful in differentiating positive and negative discriminating features in the context of imbalanced data.^37^ These are calculated based on the mean change in error rates computed only for observations belonging to a specific class. Finally, we present Shapley Additive Explanations^38,39^ in eFigures 2 to 7 in the Supplement. These Shapley Additive Explanations help to further identify the specific values of important features that “push” a prediction toward a 0 or 1.

## Results

### Descriptive Statistics

There are a total of 4 976 391 transactions in the DROS database (1996-2015) representing 1 951 006 individuals (1 525 754 men [78.2% of individuals]; mean [SD] age, 43.4 [13.9] years). Within 1 year of purchase, 2614 unique individual purchasers representing a total of 3278 transactions (0.07% of total transactions) died by firearm suicide.

Table 1 compares key features for transactions associated with individuals who died by firearm suicide within 1 year of purchase and those who did not. Differences of note for transactions involving persons who died by firearm suicide include a larger fraction of revolver purchases among those who died by firearm suicide (36.4% vs 19.3%), older mean (SD) age (47.3 [16.9] vs 43.4 [13.9] years), a higher proportion female (13.6% vs 8.5%), a higher proportion White (77.8% vs 69.5%), and fewer mean (SD) prior firearm purchases in the previous 10 years (1.6 [4.3] vs 4.9 [13.3]). Those who died by firearm suicide had fewer handgun transactions; 69.5% of that group (683 of 983) vs 39.3% of other purchasers (585 778 of 1 491 933) had no prior transaction.

**Table 1.** Transaction and Individual Features Associated With Individuals Who Died by Firearm Suicide Within 1 Year of Purchase and Other Purchasers^a^

Feature	Transactions, No. (%)	
	Associated with individual who died by firearm suicide within 1 y	Associated with other purchasers
No. (%) with data	3278 (0.07)	4 973 113 (99.9)
Firearm information		
Caliber		
Small	371 (11.3)	655 295 (13.2)
Medium	1448 (44.2)	1 826 791 (36.7)
Large	1286 (39.2)	2 236 376 (45.0)
Firearm category		
Semiautomatic	1455 (44.4)	2 964 867 (59.6)
Revolver	1192 (36.4)	958 536 (19.3)
Inexpensive make	139 (4.2)	142 382 (2.9)
Transaction information		
Gun show	43 (1.3)	97 672 (2.0)
Transaction was denied	104 (3.2)	118 763 (2.4)
Purchase month		
June	294 (9.0)	387 739 (7.8)
December	278 (85)	501 126 (10.1)
Purchaser information		
Distance to firearms dealer, km		
Mean (SD)	52.1 (137.6)	53.3 (126.5)
Lowest octile (0-3.57)^b^	553 (16.9)	621 007 (12.5)
Highest octile (≥72)	336 (10.3)	621 224 (12.5)
Female	446 (13.6)	424 806 (8.5)
Age, mean (SD), y	47.3 (16.9)	43.4 (13.9)
State of birth, California	1510 (46.1)	2 703 374 (54.4)
White race^c^	2551 (77.8)	3 458 095 (69.5)
Latino ethnicity^c^	221 (6.7)	653 439 (13.1)
Black race^c^	128 (3.9)	202 244 (4.1)
Transactions in the previous 10 y, mean (SD)	1.6 (4.3)	4.9 (13.3)
Transactions in the past year, mean (SD)	0.5 (1.3)	1.3 (3.3)
Census tract community characteristics (purchaser)		
Proportion with public assistance in past year, mean (SD)	0.06 (0.06)	0.05 (0.06)
Proportion non-Hispanic White, mean (SD)	0.5 (0.2)	0.5 (0.3)
Proportion below the federal poverty level, mean (SD)	0.1 (0.1)	0.1 (0.1)
Proportion aged 15-34 y, mean (SD)		
Female	0.1 (0.04)	0.1 (0.04)
Male	0.2 (0.1)	0.1 (0.1)
Additional variables		
RUC codes, most urban (purchaser)	2570 (78.4)	3 484 040 (70.1)
Statewide No. of handgun purchases past 12 mo, mean (SD)	21 209 (8892.5)	23 251 (9614.3)

Abbreviation: RUC, Rural-Urban Continuum.

^a^ The table presents select features and categories included in the model. Additional community characteristics are also presented in the eTable in the Supplement.

^b^ The distance traveled is uniform across octiles among nonvictims; among victims, almost half (47.2% [464 of 983]) traveled less than 10 km (the bottom 3 octiles).

^c^ The most common categories for race and ethnicity are shown. The model included a single categorical variable with all 19 race and ethnicity categories that are available in DROS (American Indian, Asian, Asian Indian, Black, Cambodian, Chinese, Filipino, Guamanian, Hawaiian, Hispanic, Japanese, Korean, Laotian, Pacific Islander, Samoan, Vietnamese, White, other, and unknown).

### Prediction of Firearm Suicide

The training set contained 3 483 475 transactions and 2295 firearm suicides (0.07%); the test set was the remaining 1 492 916 transactions, which included 983 firearm suicides (0.07%). The final algorithm built with our training data randomly selected 10 predictor variables at each split and contained 1001 trees. All performance metrics are reported on the test data.

Our algorithm AUC is 0.81 (95% CI, 0.80-0.83); the AUC-PR is 0.03. The default threshold (0.50) results in a sensitivity of 0.50 and specificity of 0.90 (Table 2). A threshold of 0.38 optimizes the Youden index and yields an algorithm sensitivity of 0.75 and specificity of 0.71. The threshold that maximizes the F-score (0.90) generates extremely high specificity (0.999) but much lower sensitivity (0.04). Finally, a threshold of 0.57 would result in classifying all instances in the riskiest 5% as a firearm suicide. In this case, sensitivity is 0.39 and specificity is 0.95, with 38.6% of observed firearm suicides (379 of 983) associated with transactions classified in the highest-risk ventile. eFigure 1 in the Supplement shows the values of these metrics for thresholds ranging from 0.20 to 0.99.

**Table 2.** Algorithm Performance Metrics With Varying Thresholds

Threshold	Sensitivity	Specificity	PPV	NPV	FPR	Youden index	F-score
0.38 (Optimize Youden index)^a^	0.748	0.712	0.002	0.999	0.288	0.459	0.003
0.50 (Default)	0.503	0.903	0.003	0.999	0.097	0.406	0.007
0.57 (Highest-risk ventile)	0.386	0.950	0.005	0.999	0.050	0.335	0.010
0.90 (Optimize F-score)^b^	0.044	0.999	0.145	0.999	0.000	0.044	0.067

Abbreviations: FPR, false positive rate; NPV, negative predictive value; PPV, positive predictive value.

^a^ Youden index: (sensitivity + specificity − 1).

^b^ F-score: (2 × sensitivity × specificity)/(sensitivity + specificity).

**Figure 1.**
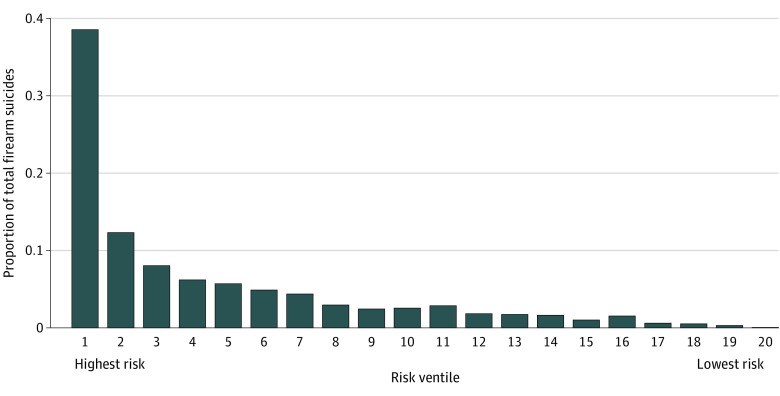
Proportion of Observed Firearm Suicides Within 1 Year of Purchase by Ventile of Predicted Risk

**Figure 2.**
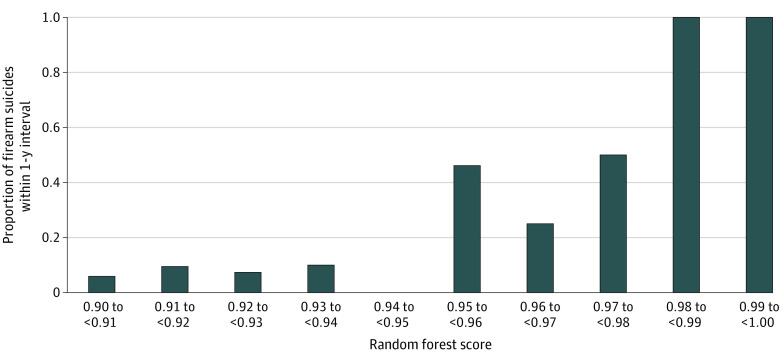
Proportion of Firearm Suicides Within 1 Year of Purchase Among High-risk Transactions

Although an RF threshold selection gives a binary response (1 if above the threshold and 0 if below the threshold), it can often be useful to examine the raw scores themselves and the likelihood of the event of interest. Figure 1 shows the predicted probabilities generated by the algorithm ranked from highest to lowest risk and grouped into equal size ventiles with the observed proportion of firearm suicides on the y-axis. Approximately half (50.9% [500 of 983]) of all firearm suicides within a year are among the top 2 ventiles of predicted risk, with a specificity of 90%. The algorithm is significantly more accurate among transactions deemed extremely risky (Figure 2). For example, more than two-thirds (24 of 35 [68.6%]) of the transactions in the test set with an RF score of 0.95 and above were associated with a purchaser who died by firearm suicide within 1 year. Among transactions with a score of 0.90 and above, 43 of 296 (14.5%) were followed by a firearm suicide within 1 year.

### Variable Importance

**Figure 3.**
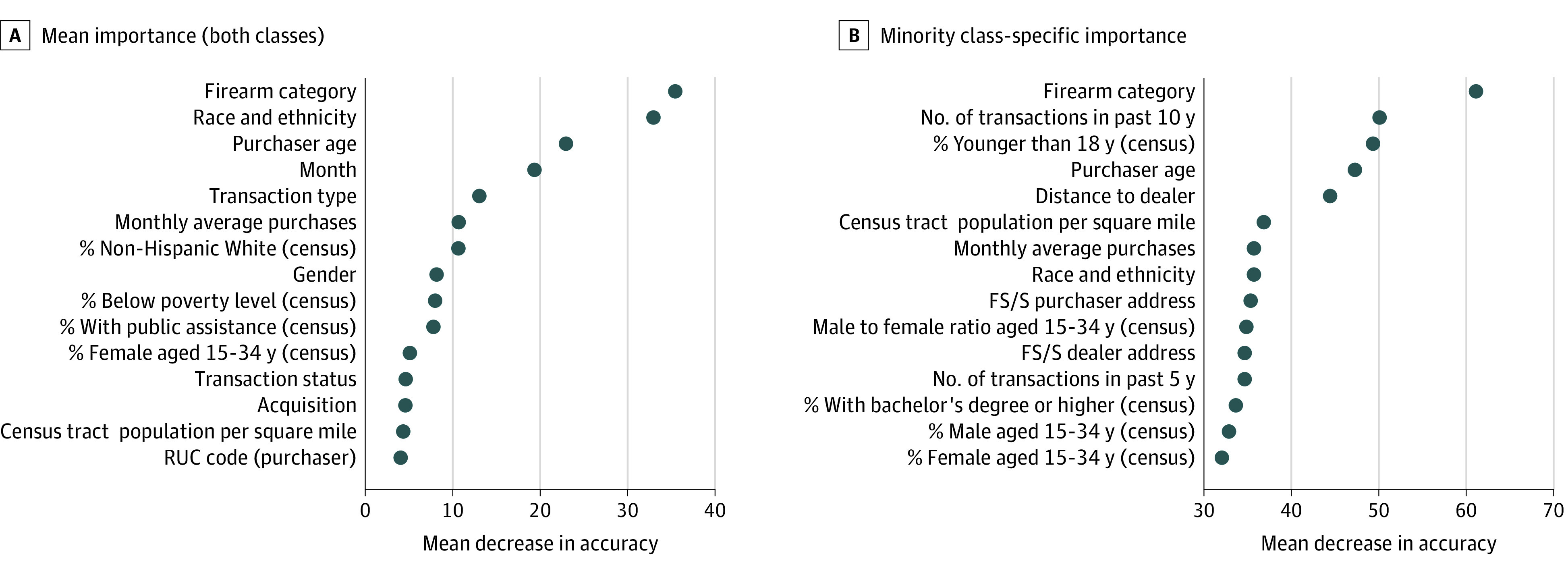
Variable Importance, Measured by Mean Decrease in Accuracy FS/S indicates firearm suicide/total suicide; RUC, Rural-Urban Continuum.

Figure 3 shows the 15 most important variables in descending importance, both overall and specific to minority class. Handgun category (revolver, semiautomatic, or other), purchaser race and ethnicity, purchaser age, and the month of the transaction are the most important features overall. Handgun category and purchaser age are also among the most minority class–specific features. Other minority class–specific important features include the number of transactions within the past 10 years, distance between the purchaser’s address and the dealer, and census tract percentage of the population younger than 18 years.

## Discussion

The present study has shown that passively collected individual-level handgun purchase data can be used to create moderately informative predictive algorithms of firearm suicide risk. Our work points to the tradeoffs and challenge of balancing true positives and false negatives. We can obtain almost perfect specificity and a much higher positive predictive value using a higher vote threshold, but the tradeoff is poor sensitivity. The desired threshold and corresponding tradeoffs depend on the purpose of the classification and the costs and benefits associated with any intervention that a risk prediction algorithm might help serve.

A range of possible interventions might be considered with an algorithm such as the one we have developed. For example, a firearm suicide risk flag for transactions identified as higher risk could prompt a gun dealer to provide suicide prevention information to purchasers, which could improve programs such as the Gun Shop Project that aims to teach gun retailers about suicide red flags and to avoid selling firearms to suicidal customers.^40,41^ With perhaps a higher threshold, a flagging system could prompt a letter from the California Department of Justice during the mandatory 10-day waiting period between purchase and pick-up of the firearm, a period that provides time for an individual to potentially seek help or reconsider suicide before gaining access to the firearm. Previous work suggests that other interventions during the 10-day waiting period can have an effect: a randomized experiment showed that a letter stating sanctions for legal violations sent to new gun buyers led to a higher rate at which guns were reported stolen among those who received the letter.^42^ There also is research to support low-cost and scalable suicide risk prevention efforts; for example, a brief caring text message sent to military service members identified as being at risk of suicide was shown to reduce rates of suicide attempt and ideation.^43^

Among the relatively few transactions deemed highest risk, a more considerable intervention might be considered. For example, for transactions with scores above 0.90 (0.02% of transactions [296 of 1 492 916]) or 0.95 (0.002% of transactions [35 of 1 492 916]), a flag for further investigation to consider the possibility of something like a civil extreme risk protection order (often known as a “red flag” order),^44^ which allows courts to remove or prevent access to firearms from those judged to be at imminent risk of violence or self-harm, could be considered. Here, the much costlier intervention would trade sensitivity for extremely high specificity and higher positive predictive value. Research suggests that civil extreme risk protection orders can be an effective suicide prevention tool, with an estimated 1 life saved for every 10-gun to 20-gun seizure actions.^44,45^

Machine learning procedures such as RF can also be useful in identifying variables that have strong predictive relationships and warrant further study.^46^ We identified previously unreported predictors of firearm suicide and confirmed the importance of several known risk factors. The type of firearm purchased was the most important predictor of firearm suicide (overall and in the minority class–specific importance measures), which is consistent with findings from a recent nested case-control study suggesting that the purchase of a revolver (vs a semiautomatic pistol) is associated with increased suicide risk.^47^ Semiautomatic handguns are more popular than revolvers,^47,48^ with features such as greater ammunition capacity that may not be important to an individual purchasing with suicidal intent. Month of purchase was an important feature overall. This finding is consistent with many studies^49^ and with California death records, which found peaks in the spring and early summer (eFigure 8 in the Supplement). Purchaser age and race and ethnicity, both known risk factors, were also among the most important features, consistent with the population-level observation that increased suicide rates are associated with age and are highest among older White men.^50^

The number of transactions in the past 10 years was the second most important minority class–specific important feature. Those who died by firearm suicide had fewer prior handgun purchases (Table 1). Distance to the firearms dealer was also an important minority class–specific feature. The data show that a larger share of purchasers who died by firearm suicide traveled a shorter distance compared with other purchasers (Table 1) and shorter distance traveled was associated with an increased probability of a positive classification (eFigure 5 in the Supplement). This finding may reflect the geographic location of individuals who died by firearm suicide, a difference in the inclination to travel, or less-selective shopping among those purchasing with suicidal intent. Research suggests that regulating alcohol outlet density can be a useful public health tool for reducing consumption and related harms.^51^ Further research might investigate the association between firearm dealer proximity and firearm acquisition and subsequent harms.

### Limitations

This study has some limitations. Although our algorithm shows promise, there remains significant opportunity to improve predictive performance. We used only administrative predictors available or generated from handgun purchasing records. Adding data with indicators of substance use or mental health disorders, for example, could potentially generate much more powerful prediction. Future work might also consider the development of a second-stage algorithm among those deemed higher risk, as has been recently proposed.^52^ Other classification algorithms (eg, support vector machines^53^ and logistic regression)^54^ or ensemble methods (eg, as super learner)^55^ could also be explored to improve predictive accuracy, as might unsupervised anomaly detection approaches.^56^ Additionally, our algorithm is at the transaction level; future work should consider individual-level survival risk prediction or a repeated-measures RF approach.^57^

There are also a number of data limitations. The DROS records contain missing and inconsistent values; these, along with imperfect linking and geocoding processes, introduce error and noise. We do not have records for illegal firearm acquisitions or long gun acquisitions. The outcome data on firearm suicide do not include out-of-state deaths for nonresidents.

In addition, the analyses are limited to handgun purchasers; during the study period, 38% of individuals who died by firearm suicide in California had at least 1 record in DROS. Even among handgun purchasers, many of the firearm suicides occur among individuals classified as low risk. Other forms of intervention would be necessary to prevent firearm suicide among this group.

Finally, only 11 states currently require licensed dealers to report identified firearm sales information to law enforcement.^58^ Risk prediction such as we have demonstrated here is not possible in other states.

## Conclusions

Accurate suicide risk prediction can play an important role in lethal means interventions. Evidence suggests that the vast majority of people who survive a suicide attempt do not go on to die by suicide,^58^ and policies that restrict firearm access among high-risk groups can be an effective means of preventing firearm suicide.^59^ This study contributes to the growing evidence that computational methods can aid in the identification of high-risk groups and the development of targeted interventions. This study suggests that handgun transaction information routinely collected in California can be used to create predictive algorithms of firearm suicide risk.

## Notes

Supplement.
eMethods.
**eFigure 1.** Random Forest Performance Metrics With Varying Thresholds, Firearm Suicide Within 1 Year of Transaction **eFigure 2.** SHAP Feature Contribution (Top 15) **eFigure 3.** SHAP Violin Summary Plot: Firearm Category **eFigure 4.** SHAP Violin Summary Plot: Number of Transactions in Past 10 Years **eFigure 5.** SHAP Violin Summary Plot: Distance to Dealer **eFigure 6.** SHAP Violin Summary Plot: Purchaser Age **eFigure 7.** SHAP Violin Summary Plot: Proportion of the Population Under 18 **eFigure 8.** Monthly Trends in Firearm Suicides **eTable.** Additional Community Characteristics (Transactions Associated With Individuals Who Died by Firearm Suicide Within 1 Year)
eReferences.

## References

[1] Centers for Disease Control and Prevention. WISQARS—Web-based Injury Statistics Query and Reporting System: fatal injury data. 2021. Accessed March 2022. https://www.cdc.gov/injury/wisqars/fatal.html

[2] Elnour AA, Harrison J. Lethality of suicide methods. Inj Prev. 2008;14(1):39-45. doi:10.1136/ip.2007.016246 18245314

[3] Conner A, Azrael D, Miller M. Suicide case-fatality rates in the United States, 2007 to 2014: a nationwide population-based study. Ann Intern Med. 2019;171(12):885-895. doi:10.7326/M19-1324 31791066

[4] Anglemyer A, Horvath T, Rutherford G. The accessibility of firearms and risk for suicide and homicide victimization among household members: a systematic review and meta-analysis. Ann Intern Med. 2014;160(2):101-110. doi:10.7326/M13-1301 24592495

[5] Barber CW, Miller MJ. Reducing a suicidal person’s access to lethal means of suicide: a research agenda. Am J Prev Med. 2014;47(3)(suppl 2):S264-S272. doi:10.1016/j.amepre.2014.05.028 25145749

[6] Studdert DM, Zhang Y, Swanson SA, . Handgun ownership and suicide in California. N Engl J Med. 2020;382(23):2220-2229. doi:10.1056/NEJMsa1916744 32492303

[7] Cummings P, Koepsell TD, Grossman DC, Savarino J, Thompson RS. The association between the purchase of a handgun and homicide or suicide. Am J Public Health. 1997;87(6):974-978. doi:10.2105/AJPH.87.6.974 9224179 PMC1380933

[8] Franklin JC, Ribeiro JD, Fox KR, . Risk factors for suicidal thoughts and behaviors: a meta-analysis of 50 years of research. Psychol Bull. 2017;143(2):187-232. doi:10.1037/bul0000084 27841450

[9] Jordan MI, Mitchell TM. Machine learning: trends, perspectives, and prospects. Science. 2015;349(6245):255-260. doi:10.1126/science.aaa8415 26185243

[10] Walsh CG, Ribeiro JD, Franklin JC. Predicting suicide attempts in adolescents with longitudinal clinical data and machine learning. J Child Psychol Psychiatry. 2018;59(12):1261-1270. doi:10.1111/jcpp.12916 29709069

[11] Simon GE, Johnson E, Lawrence JM, . Predicting suicide attempts and suicide deaths following outpatient visits using electronic health records. Am J Psychiatry. 2018;175(10):951-960. doi:10.1176/appi.ajp.2018.17101167 29792051 PMC6167136

[12] Barak-Corren Y, Castro VM, Nock MK, . Validation of an electronic health record–based suicide risk prediction modeling approach across multiple health care systems. JAMA Netw Open. 2020;3(3):e201262. doi:10.1001/jamanetworkopen.2020.1262 32211868 PMC11136522

[13] Kessler RC, Hwang I, Hoffmire CA, . Developing a practical suicide risk prediction model for targeting high-risk patients in the Veterans Health Administration. Int J Methods Psychiatr Res. 2017;26(3):e1575. doi:10.1002/mpr.1575 28675617 PMC5614864

[14] Kessler RC, Warner CH, Ivany C, ; Army STARRS Collaborators. Predicting suicides after psychiatric hospitalization in US Army soldiers: the Army Study To Assess Risk and Resilience in Servicemembers (Army STARRS). JAMA Psychiatry. 2015;72(1):49-57. doi:10.1001/jamapsychiatry.2014.1754 25390793 PMC4286426

[15] Roy A, Nikolitch K, McGinn R, Jinah S, Klement W, Kaminsky ZA. A machine learning approach predicts future risk to suicidal ideation from social media data. NPJ Digit Med. 2020;3(1):78. doi:10.1038/s41746-020-0287-6 32509975 PMC7250902

[16] Bossarte RM, Kennedy CJ, Luedtke A, . Invited commentary: new directions in machine learning analyses of administrative data to prevent suicide-related behaviors. Am J Epidemiol. 2021;190(12):2528-2533. doi:10.1093/aje/kwab111 33877322 PMC8796802

[17] McHugh CM, Large MM. Can machine-learning methods really help predict suicide? Curr Opin Psychiatry. 2020;33(4):369-374. doi:10.1097/YCO.0000000000000609 32250986

[18] Collins GS, Reitsma JB, Altman DG, Moons KG. Transparent reporting of a multivariable prediction model for individual prognosis or diagnosis (TRIPOD): the TRIPOD statement. Br J Surg. 2015;102(3):148-158. doi:10.1002/bjs.9736 25627261

[19] Cal Penal Code §27505. Accessed June 5, 2022. https://leginfo.legislature.ca.gov/faces/codes_displaySection.xhtml?lawCode=PEN&sectionNum=27505

[20] Fjestad SP, Fjestad SP. Blue Book of Gun Values. Blue Book Publications; 2017.

[21] Azrael D, Cook PJ, Miller M. State and local prevalence of firearms ownership measurement, structure, and trends. J Quant Criminol. 2004;20(1):43-62. doi:10.1023/B:JOQC.0000016699.11995.c7

[22] Breiman L. Random forests. Mach Learn. 2001;45(1):5-32. doi:10.1023/A:1010933404324

[23] Fernández-Delgado M, Cernadas E, Barro S, Amorim D. Do we need hundreds of classifiers to solve real world classification problems? J Mach Learn Res. 2014;15(1):3133-3181.

[24] Muchlinski D, Siroky D, He J, Kocher M. Comparing random forest with logistic regression for predicting class-imbalanced civil war onset data. Polit Anal. 2016;24(1):87-103. doi:10.1093/pan/mpv024

[25] Wheeler AP, Steenbeek W. Mapping the risk terrain for crime using machine learning. J Quant Criminol. 2021;37(2):445-480. doi:10.1007/s10940-020-09457-7

[26] Berk R, Sherman L, Barnes G, Kurtz E, Ahlman L. Forecasting murder within a population of probationers and parolees: a high stakes application of statistical learning. J R Stat Soc Ser A Stat Soc. 2009;172(1):191-211. doi:10.1111/j.1467-985X.2008.00556.x

[27] Khalilia M, Chakraborty S, Popescu M. Predicting disease risks from highly imbalanced data using random forest. BMC Med Inform Decis Mak. 2011;11(1):51. doi:10.1186/1472-6947-11-51 21801360 PMC3163175

[28] Hastie T, Tibshirani R, Friedman JH, Friedman JH. The Elements of Statistical Learning: Data Mining, Inference, and Prediction. Vol 2. Springer; 2009. doi:10.1007/978-0-387-84858-7

[29] He H, Garcia EA. Learning from imbalanced data. IEEE Trans Knowl Data Eng. 2009;21(9):1263-1284. doi:10.1109/TKDE.2008.239

[30] Kuhn M, Johnson K. Applied Predictive Modeling. Vol 26. Springer; 2013. doi:10.1007/978-1-4614-6849-3

[31] Singh A, Ranjan RK, Tiwari A. Credit card fraud detection under extreme imbalanced data: a comparative study of data-level algorithms. J Exp Theor Artif Intell. Published online April 3, 2021. doi:10.1080/0952813X.2021.1907795

[32] Kuhn M, Wing J, Weston S, ; R Core Team. Package ‘caret.’ Classification and Regression Training. April 19, 2022. Accessed June 7, 2022. https://cran.r-project.org/web/packages/caret/caret.pdf

[33] Liaw A, Wiener M. Classification and regression by randomForest. R News. 2002;2(3):18-22.

[34] He H, Ma Y, eds. Imbalanced Learning: Foundations, Algorithms, and Applications. Wiley-IEEE Press; 2013. doi:10.1002/9781118646106

[35] Sokolova M, Japkowicz N, Szpakowicz S. Beyond accuracy, F-score and ROC: a family of discriminant measures for performance evaluation. In: *Australasian Joint Conference on Artificial Intelligence*. Springer; December 2006:1015-1021.

[36] Martínez-Camblor P, Pardo-Fernández JC. The Youden index in the generalized receiver operating characteristic curve context. Int J Biostat. 2019;15(1):20180060. doi:10.1515/ijb-2018-0060 30943172

[37] Vigil A. Building Explainable Random Forest Models With Applications in Protein Functional Analysis. San Francisco State University; 2016.

[38] Lundberg SM, Lee SI. A unified approach to interpreting model predictions. Preprint posted online May 22, 2017. doi:10.48550/arXiv.1705.07874

[39] Greenwell B, Greenwell MB. Package ‘fastshap: Fast Approximation Shapley Values.’ R package. Version 0.0.7. 2021. Accessed May 15, 2022. https://cran.r-project.org/web/packages/fastshap/fastshap.pdf

[40] Walton T, Stuber J. Firearm retailers and suicide: results from a survey assessing willingness to engage in prevention efforts. Suicide Life Threat Behav. 2020;50(1):83-94. doi:10.1111/sltb.12574 31355478

[41] Allchin A, Chaplin V, Horwitz J. Limiting access to lethal means: applying the social ecological model for firearm suicide prevention. Inj Prev. 2019;25(suppl 1):i44-i48. doi:10.1136/injuryprev-2018-042809 29941633

[42] Ridgeway G, Braga AA, Tita G, Pierce GL. Intervening in gun markets: an experiment to assess the impact of targeted gun-law messaging. J Exp Criminol. 2011;7(1):103-109. doi:10.1007/s11292-010-9113-5

[43] Comtois KA, Kerbrat AH, DeCou CR, . Effect of augmenting standard care for military personnel with brief caring text messages for suicide prevention: a randomized clinical trial. JAMA Psychiatry. 2019;76(5):474-483. doi:10.1001/jamapsychiatry.2018.4530 30758491 PMC6495345

[44] Swanson JW. Preventing suicide through better firearm safety policy in the United States. Psychiatr Serv. 2021;72(2):174-179. doi:10.1176/appi.ps.202000317 32878544

[45] Swanson JW, Norko MA, Lin HJ, . Implementation and effectiveness of Connecticut’s risk-based gun removal law: does it prevent suicides? Law Contemp Probl. 2017;80:179-208.

[46] Shmueli G. To explain or to predict? Stat Sci. 2010;25(3):289-310. doi:10.1214/10-STS330

[47] Schleimer JP, Kagawa RMC, Laqueur HS. Handgun purchasing characteristics and firearm suicide risk: a nested case-control study. Inj Epidemiol. 2021;8(1):68. doi:10.1186/s40621-021-00365-3 34903267 PMC8666831

[48] Kravitz-Wirtz N, Pallin R, Miller M, Azrael D, Wintemute GJ. Firearm ownership and acquisition in California: findings from the 2018 California Safety and Well-being Survey. Inj Prev. 2020;26(6):516-523. doi:10.1136/injuryprev-2019-043372 31806674

[49] Christodoulou C, Efstathiou V, Bouras G, Korkoliakou P, Lykouras L. Seasonal variation of suicide: a brief review. Encephalos. 2012;49(73):9.

[50] Riddell CA, Harper S, Cerdá M, Kaufman JS. Comparison of rates of firearm and nonfirearm homicide and suicide in Black and White non-Hispanic men, by US state. Ann Intern Med. 2018;168(10):712-720. doi:10.7326/M17-2976 29710093

[51] Campbell CA, Hahn RA, Elder R, et al. The effectiveness of limiting alcohol outlet density as a means of reducing excessive alcohol consumption and alcohol-related harms. Am J Prev Med. 2009;37(6):556-569. doi:10.1016/j.amepre.2009.09.028 19944925

[52] Kessler RC, Bossarte RM, Luedtke A, Zaslavsky AM, Zubizarreta JR. Suicide prediction models: a critical review of recent research with recommendations for the way forward. Mol Psychiatry. 2020;25(1):168-179. doi:10.1038/s41380-019-0531-0 31570777 PMC7489362

[53] Hearst MA, Dumais ST, Osuna E, Platt J, Scholkopf B. Support vector machines. IEEE Intell Syst Their Appl. 1998;13(4):18-28. doi:10.1109/5254.708428

[54] Dreiseitl S, Ohno-Machado L. Logistic regression and artificial neural network classification models: a methodology review. J Biomed Inform. 2002;35(5-6):352-359. doi:10.1016/S1532-0464(03)00034-0 12968784

[55] van der Laan MJ, Polley EC, Hubbard AE. Super learner. Stat Appl Genet Mol Biol. 2007;6(1):e25. doi:10.2202/1544-6115.1309 17910531

[56] Carcillo F, Le Borgne YA, Caelen O, Kessaci Y, Oblé F, Bontempi G. Combining unsupervised and supervised learning in credit card fraud detection. Inf Sci. 2021;557:317-331. doi:10.1016/j.ins.2019.05.042

[57] Calhoun P, Levine RA, Fan J. Repeated measures random forests (RMRF): identifying factors associated with nocturnal hypoglycemia. Biometrics. 2021;77(1):343-351. doi:10.1111/biom.13284 32311079

[58] Giffords Law Center to Prevent Gun Violence. Maintaining records of gun sales. Published 2018. Accessed December 2, 2020. https://lawcenter.giffords.org/gun-laws/policy-areas/gun-sales/maintaining-records-of-gun-sales/

[59] Suominen K, Isometsä E, Suokas J, Haukka J, Achte K, Lönnqvist J. Completed suicide after a suicide attempt: a 37-year follow-up study. Am J Psychiatry. 2004;161(3):562-563. doi:10.1176/appi.ajp.161.3.562 14992984

