# Supplementary material for: Machine Learning Analysis of Handgun Transactions to Predict Firearm Suicide Risk
Source: JAMA Netw Open. 2022 Jul 11;5(7):e2221041. doi: 10.1001/jamanetworkopen.2022.21041 (PMC9274320; doi:10.1001/jamanetworkopen.2022.21041)
Supplement: Supplement. — eMethods. eFigure 1. Random Forest Performance Metrics With Varying Thresholds, Firearm Suicide Within 1 Year of Transaction eFigure 2. SHAP Feature Contribution (Top 15) eFigure 3. SHAP Violin Summary Plot: Firearm Category eFigure 4. SHAP Violin Summary Plot: Number of Transactions in Past 10 Years eFigure 5. SHAP Violin Summary Plot: Distance to Dealer eFigure 6. SHAP Violin Summary Plot: Purchaser Age eFigure 7. SHAP Violin Summary Plot: Proportion of the Population Under 18 eFigure 8. Monthly Trends in Firearm Suicides eTable. Additional Community Characteristics (Transactions Associated With Individuals Who Died by Firearm Suicide Within 1 Year) eReferences. [file jamanetwopen-e2221041-s001.pdf]

Supplementary Online Content

Laqueur HS, Smirniotis C, McCort C, Wintemute GJ. Machine learning analysis of handgun transactions to predict firearm suicide risk. *JAMA Netw Open.* 2022;5(7):e2221041. doi:10.1001/jamanetworkopen.2022.21041

eMethods.
eFigure 1. Random Forest Performance Metrics With Varying Thresholds, Firearm Suicide Within 1 Year of Transaction
eFigure 2. SHAP Feature Contribution (Top 15)
eFigure 3. SHAP Violin Summary Plot: Firearm Category
eFigure 4. SHAP Violin Summary Plot: Number of Transactions in Past 10 Years
eFigure 5. SHAP Violin Summary Plot: Distance to Dealer
eFigure 6. SHAP Violin Summary Plot: Purchaser Age
eFigure 7. SHAP Violin Summary Plot: Proportion of the Population Under 18
eFigure 8. Monthly Trends in Firearm Suicides
eTable. Additional Community Characteristics (Transactions Associated With Individuals Who Died by Firearm Suicide Within 1 Year)
eReferences.

This supplementary material has been provided by the authors to give readers additional information about their work.

© 2022 Laqueur HS et al. *JAMA Network Open.*

eMethods.

Here we provide a full description of the data and analytic approach. An abbreviated description of the methods is provided in the manuscript.

Data

Our primary predictive variables were generated from DROS transaction records for handgun transfers (January 1, 1996 to October 6, 2015). This consists of 4,976,391 records for 1,951,006 individuals. DROS records are maintained in the California Department of Justice (CA DOJ) Automated Firearms System (AFS) database. Under California law, essentially all transfers of firearms must be done through a licensed firearms retailer, including transfers between private parties, gun show sales, gifts, loans, and the redemption of pawned or consigned weapons. The legal age to purchase a handgun in California is 21. Detailed records for handgun transactions have been recorded since 1996; record-keeping for long guns only began in 2014, thus we restricted our analyses to handguns. The DROS records include purchaser identifiers, address, the date and time of the transaction, identifiers for the seller, and the caliber, type, make and model of the firearm.[1]

Outcome Measure

We identified firearm suicide deaths (*International Classification of Diseases, 9th edition and International Classification of Diseases and Related Health Problems*, 10th edition, codes X72-X74) by linking purchasers in DROS to the California Department of Public Health's Death Statistical Master File (1996–2016), using probabilistic matching on name, date of birth, and gender. Our primary model forecasts firearm suicide within 1 year of the transaction. The California Death Statistical Mater file includes cause of death, with International Statistical Classification of Diseases and Related Health Problems, Tenth Revision (ICD–10) underlying cause-of-death codes, with suicide by firearm given by codes X72–X74. While the DROS database is at the transaction level, each individual in DROS is assigned a unique identifier ("person key") that allows for linkage across transactions. We linked purchasers in DROS to the death files by probabilistic matching performed with Link Plus (version 2.0) linkage software using name and date of birth variables. Records were blocked on Soundex (a phonetic algorithm for indexing names by sound, as pronounced in English) of first name and last name. This probabilistic matching allows for partial matches, misspellings, hyphenations, typographical errors, variation in name order, names that sound similar, and nicknames. We developed a series rules for accepting matches, for example: first name, middle name, last name and birth-date match, with at least 2 exact matches; matches with an exact match on first name, middle name, and birthday, and a female gender (to pick up instances in which maiden name appeared to have been dropped), etc.

Transactions without a person key and/or date of birth were dropped from the analysis (n = 1,394). Transactions in which the date of purchase was after the date of death were dropped (n = 3,031). We also eliminated 18 transactions that were known test cases in the DROS database.

Predictor Variables

We generated 41 predictor variables from the DROS transaction data. A number of these features related to the handgun itself. These included handgun category (revolver vs. semiautomatic pistol vs. unknown); caliber, categorized into small (e.g. .22, .25, .32), medium (e.g. .38, 9 mm), and large (e.g. .40, .44, .45) and "other" which included frame only, interchangeable, missing, or any caliber not captured in our small, medium, large schema. We included an indicator for "inexpensive" handgun, proxied by the manufacturer, selecting the bottom quartile of median prices found in the *Blue Book of Gun Values*.[2]

Transaction characteristics included an indicator for whether the handgun was purchased at a gun show (yes, no, or unknown), the transaction type (e.g. sale, voluntary registration, pawn redemption, or law enforcement acquisition) and transaction status (approved, administrative denial, cancelled). We also calculated the number of purchases an individual had made in the prior 1, 2, 5, and 10 years. To calculate prior transactions, we used AFS transaction data dating back to 1985. These transaction records include personal identifiers but do not have most of the details available in DROS 1996 forward, and thus were only utilized for the purpose of calculating prior purchasing.

© 2022 Laqueur HS et al. *JAMA Network Open.*

We included individual purchaser demographic variables available in DROS. These included sex, race/ethnicity, state of birth (California vs. elsewhere), and age at the time of the transaction.

We geocoded the purchaser and dealer addresses recorded in DROS to identify the associated census tracts and obtain community characteristics. All addresses (for the purchaser and dealer) were geocoded using a multistage process to find the highest resolution match available using SAS® software, Census Bureau Geocoder, and Google Geocoding API.[3-5] (These approaches use different geodetic datums, but the magnitude of difference is not relevant for our spatial unit of analysis.) There were three sets of addresses considered: the purchaser's address as listed on the transaction data, the dealer's address, and the address contained in the DROS Person Table (a table in DROS that includes the most recent address for each person key).

If a transaction was missing the purchaser address (3.7%), we attempted to complete the information using an address from one of the following methods (in order): another transaction by the purchaser on the same day (0.067%); another transaction from a different day, closest in time to the purchase in question (2.1%); or the person table address (1.53%). Occasionally, the geocoding programs returned different coordinates for repeated instances of the same address. In these cases, if the matched latitude or longitude coordinates differed by more than 0.001 degree, the finest resolution match was used, or the first one in chronological order if tied. We manually fixed a very small percentage of addresses (less than 0.001%) that remained unmatched by all three geocoders or were assigned an out of state match. For these transactions, an internet search of the recorded address revealed a compelling alternative, such as a small typographical error in the zip code field.

There were 1,322 purchaser addresses that could not be geocoded. These included 105 known military addresses such as ships or bases that do not correspond to ACS census information, out of state addresses (less than 0.001%), and transactions with incomplete purchaser address information. All were coded as Unknown for location-based variables.

There were 2,590 transactions with no geocoding match for the dealer's address, in all but one case because the transactions had no dealer identification number associated with the transaction. In one instance, the dealer address was missing. In the person table, a unique person identifier may have more than one distinct address listed separately as home, mailing, business, or physical addresses. When this occurred, the home address was used.

Census-tract level features were obtained from Geolytics' Neighborhood Change Database, which produces US Census data normalized to 2010 tract boundaries.[6] We included a number of census tract variables from the U.S. Census and American Community Survey: population density, proportion of the population under 18, the ratio of males to females aged 15-34 years, proportion non-Latino white and non-Latino Black, the proportion of the population with past-year income below the poverty level, proportion with public assistance within the last year, the unemployment rate, and the proportion of households with children that were female-headed. Based on the geocoded address, we also identified addresses that were known military bases and created a "military address" indicator variable.

Finally, we included several county-level community characteristics associated with the purchaser's address and dealer's location. Using the Death Statistical Masterfile, we generated a county-level ratio of firearm suicides divided by total suicides (a common proxy for firearm prevalence). We also included the average suicide rate in the county over the twelve months prior to the month of the transaction. We included an estimate of local firearm purchasing trends with a moving monthly average of the number of handguns purchased (in DROS) in the county over the previous 12 months. We calculated the distance between the purchaser's address and the location of the dealer. Lastly, for both the purchaser and dealer address, we included rural-urban status based on the USDA's Rural-Urban Continuum Codes (RUC). Here too, all county-level continuous variables were converted into categorical variables by octiles, and missing values were coded as "Unknown."

We converted all continuous variables into categorical variables based on octiles, with missing values coded as "Unknown."

© 2022 Laqueur HS et al. *JAMA Network Open.*

Algorithm Development

Random forest

We implement random forest classification on the transaction-level data to predict firearm suicide within 1 year.[7] Random forest has been shown to be among the strongest performing classifiers[8] and to frequently perform well on imbalanced data.[9] It has been successfully applied in a range of contexts, including criminal offending,[10,11] disease forecasting,[12] and predicting suicide ideation.[13]

In brief, random forest works by aggregating many hundreds of classification trees, each of which represents a recursive partitioning of the training data. Each classification tree is built from a bootstrapped sample from the training data. The tree creates binary splits based on a sample of predictor variables, drawn randomly at each partition, and selecting the best (i.e. purest or most homogeneous) split. The tree is grown, without pruning, until either purity or node size 1 is reached. Each tree then predicts the outcome value for any observations in the training set that had not been used to create that tree and calculates the out-of-bag (OOB) error rate. Finally, the classification trees are aggregated to create the random forest algorithm, and each observation receives a predicted probability or score (e.g. 0.20, 0.50, 0.95, etc.) based on the proportion of trees that assign it to the positive class (class 1). Depending on the task, these scores are then converted to a class label determined by the "decision threshold." The default threshold is majority rule (observations with scores above 0.50 are assigned to class 1).[14]

Though the default threshold is majority rule, this can be adjusted to meet desired objectives and improve classifier performance.[15] For example, the threshold can be moved to maximize performance metrics of interest, e.g. composite measures of sensitivity and specificity such as Youden's Index.[16] Adjusting the threshold also offers a means to account for asymmetric costs. For example, if false negatives are much more costly than false positives, a lower threshold can be selected to achieve the desired cost-ratio.

Dealing with imbalanced data

The major challenge with predicting firearm suicide is the rarity of the event, resulting in extreme class imbalance. "Under-sampling" is a well-established technique for dealing with imbalanced data, shown to improve an algorithm's ability to isolate the event signal.[17,18] This approach balances the data by decreasing the size of the majority class. We implemented under-sampling within the random forest algorithm by using a bootstrapped sample of the same size from each class (or strata) to create a balanced data-set to grow each tree. This stratified sampling alters the marginal distribution such that the observations used to grow a tree are comprised of half firearm suicides and half non-suicides. Though the algorithm is built with data approximating balanced classes, the data used to *test* the algorithm performance remains unbalanced. For each algorithm, we allocated a random sample of 70% of the data as the training set and used the remaining 30% as a test set.

Algorithm tuning

We tuned 2 random forest parameters, the number of predictor variables to randomly select at each binary split (mtry), and the number of trees in the forest (ntree), by maximizing the area under the receiver operator characteristic curve (AUC) within the caret package in R.[19,20]

Algorithm evaluation

We evaluated the algorithm with test-set AUC, sensitivity (true positive rate), specificity (true negative rate), and metrics that combine sensitivity and specificity that are commonly used on imbalanced classification problems[21]: F-score[22] (2 ∗ sensitivity ∗ specificity) / (sensitivity + specificity), and Youden's Index (sensitivity + specificity −1).[23] We also present the positive predictive value (PPV) and negative predictive value (NPV) (i.e. the proportions of positive/negative results that are true positive/negatives). We present all of these metrics for a range of random forest vote thresholds including the default (.5), the threshold that maximizes F-score, and the threshold that maximizes Youden's Index. Finally, we also present raw scores and the proportion of suicides among the highest predicted risk transactions, defined as those with a random forest score of 0.90 and above.

© 2022 Laqueur HS et al. *JAMA Network Open.*

Variable importance

To shed some light on the "black box" algorithm, we also present variable importance measures, which provide information on predictors' contributions to classification accuracy. Random forest generates two default measures of variable importance: mean decrease in accuracy (i.e. how much accuracy the algorithm loses by excluding each variable) and mean decrease in Gini impurity (i.e. how each variable contributes to the homogeneity of node splits over all trees in the forest). Given the well-established biases of Gini importance measures when predictors vary in their scale or the number of categories,[24] we focus on mean decrease in accuracy. This approach, also known as permutation importance, permutes each variable and calculates the difference in accuracy before and after permuting (averaged over all trees and normalized by the standard deviation of the difference) to give an importance measure.

In addition to the overall permutation importance, we also present class specific importance measures, which can be useful in differentiating positive and negative discriminating features in the context of imbalanced data.[25] These are calculated based on the mean change in error rates computed only for observations belonging to a specific class. We focus on the minority class (firearm suicide); given the extreme class imbalance, the overall importance and majority class importance measures are essentially the same.

Finally, to further explain the random forest predictions, we present Shapley Additive Explanations (SHAP)[26] using *fastshap: Fast Approximation Shapley Values* in R, version 0.0.7.[27] in Supplemental figures below (eFigure 2 – 7). SHAP uses the game theory concept developed by Shapley[28] to calculate the relative contribution of each feature in the prediction model to the different prediction "coalitions" (i.e. 0 or 1). We present the top fifteen SHAP features. We present select violin plots for important minority-class specific features identified using the random forest mean decrease in accuracy to help identify the values of each feature that are relevant in "pushing" the prediction towards a 0 or 1. Positive SHAP values increase the probability of a classification to the positive class (1); negative values increase the probability of a classification to the negative class (0).

© 2022 Laqueur HS et al. *JAMA Network Open*.

eFigure 1. Random Forest Performance Metrics With Varying Thresholds, Firearm Suicide Within 1 Year of Transaction

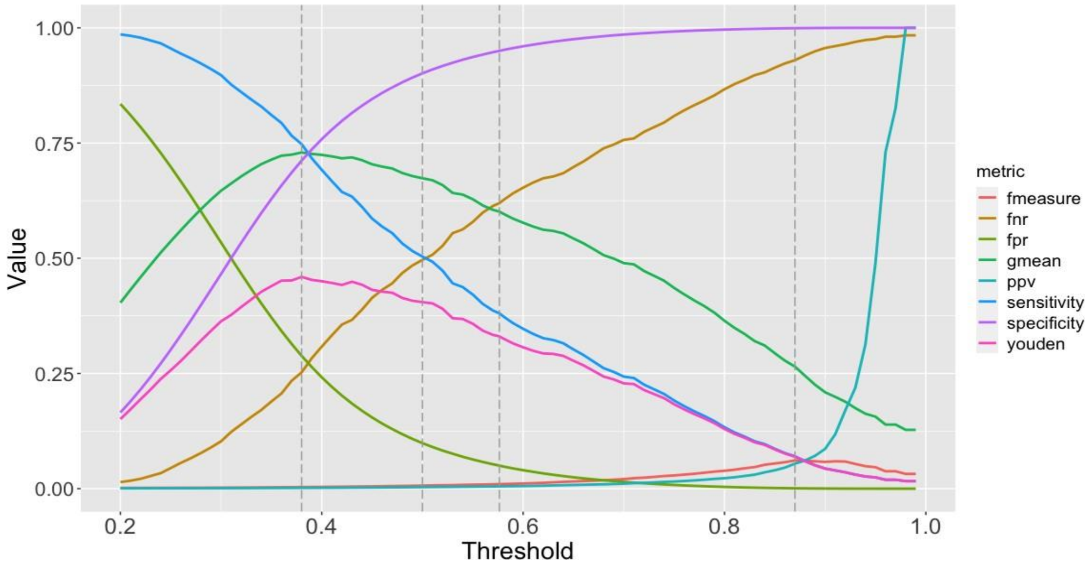

© 2022 Laqueur HS et al. *JAMA Network Open*.

eFigure 2. SHAP Feature Contribution (Top 15)

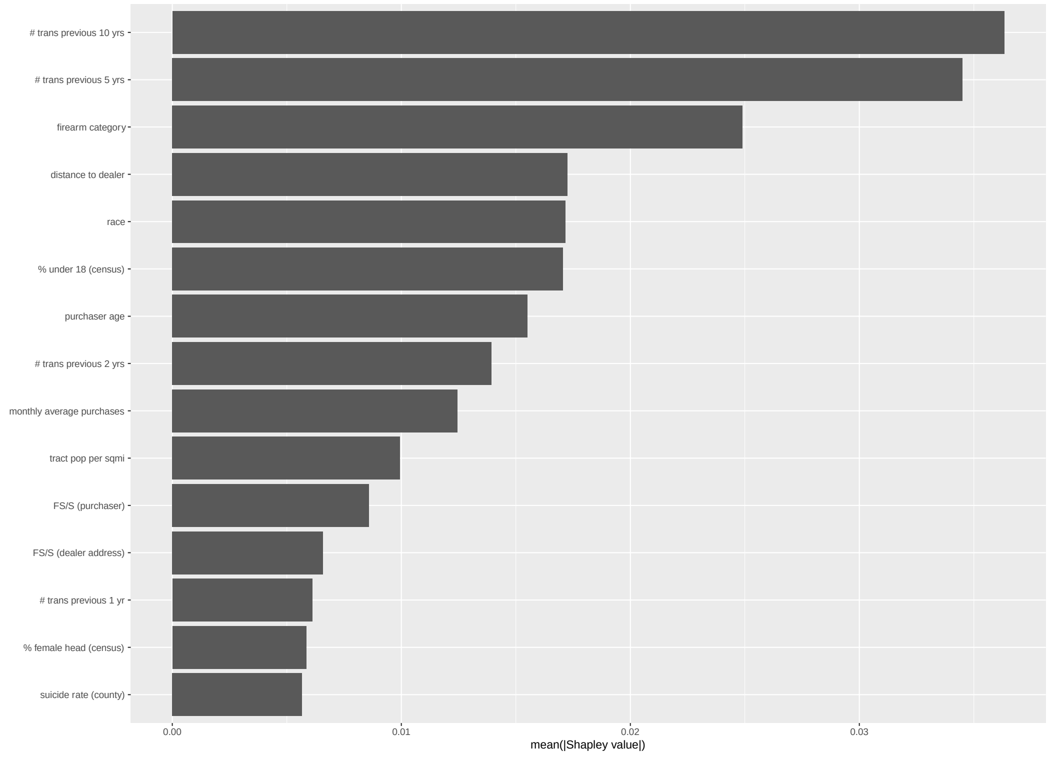

© 2022 Laqueur HS et al. *JAMA Network Open*.

eFigure 3. SHAP Violin Summary Plot: Firearm Category

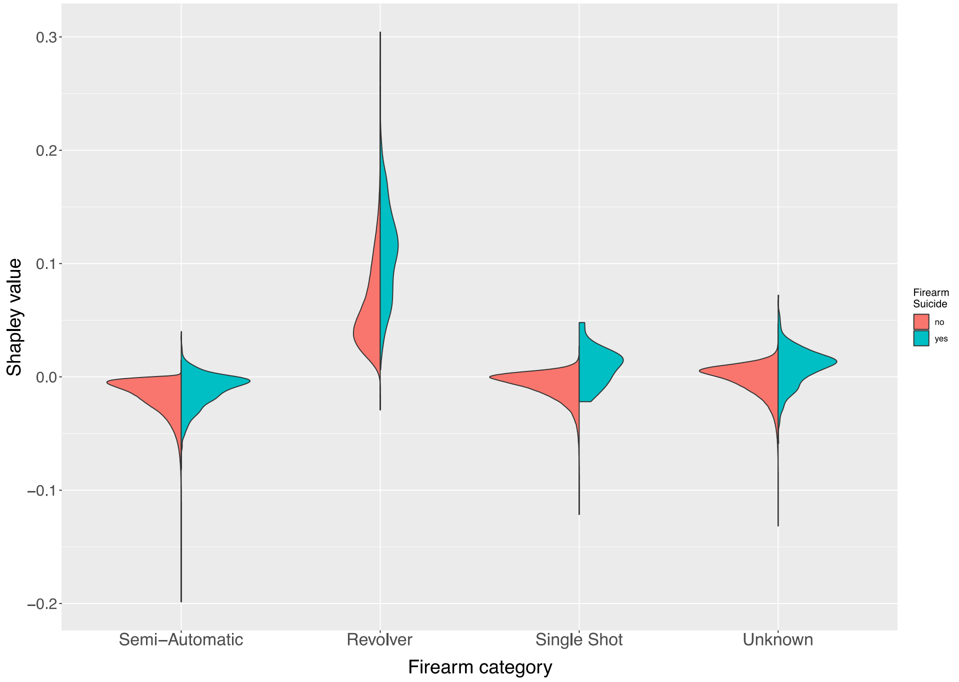

© 2022 Laqueur HS et al. *JAMA Network Open*.

eFigure 4. SHAP Violin Summary Plot: Number of Transactions in Past 10 Years

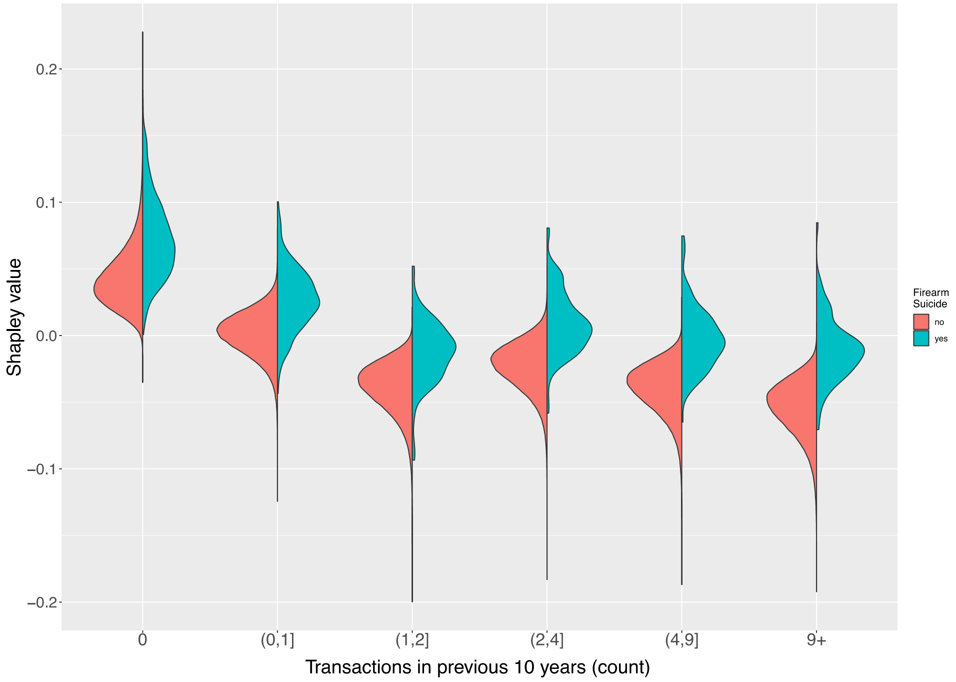

© 2022 Laqueur HS et al. *JAMA Network Open*.

eFigure 5. SHAP Violin Summary Plot: Distance to Dealer

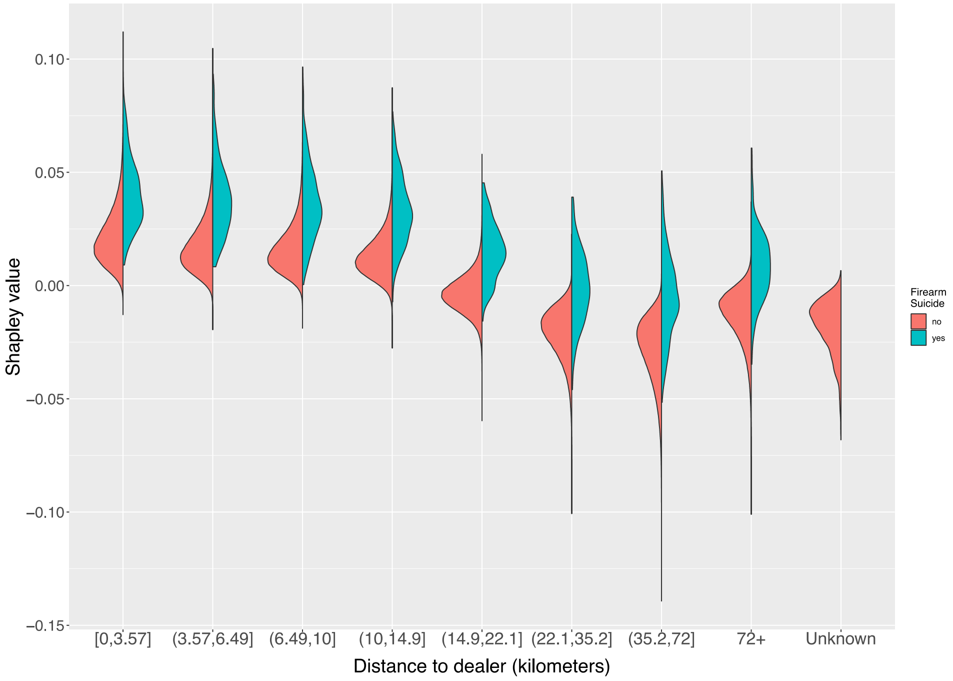

© 2022 Laqueur HS et al. *JAMA Network Open*.

eFigure 6. SHAP Violin Summary Plot: Purchaser Age

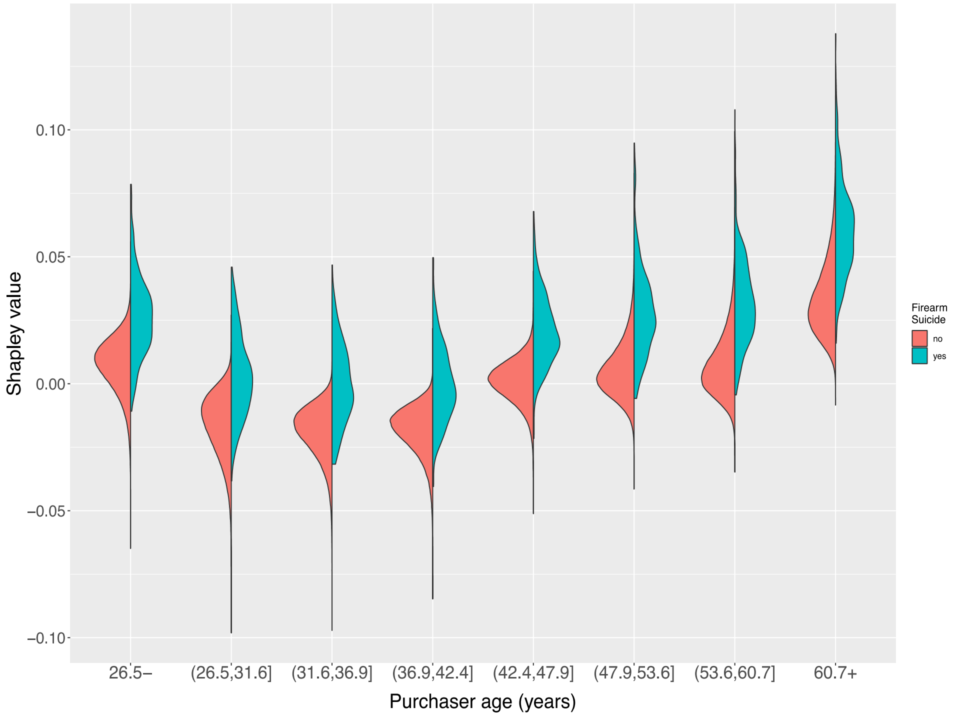

© 2022 Laqueur HS et al. *JAMA Network Open*.

eFigure 7. SHAP Violin Summary Plot: Proportion of the Population Under 18

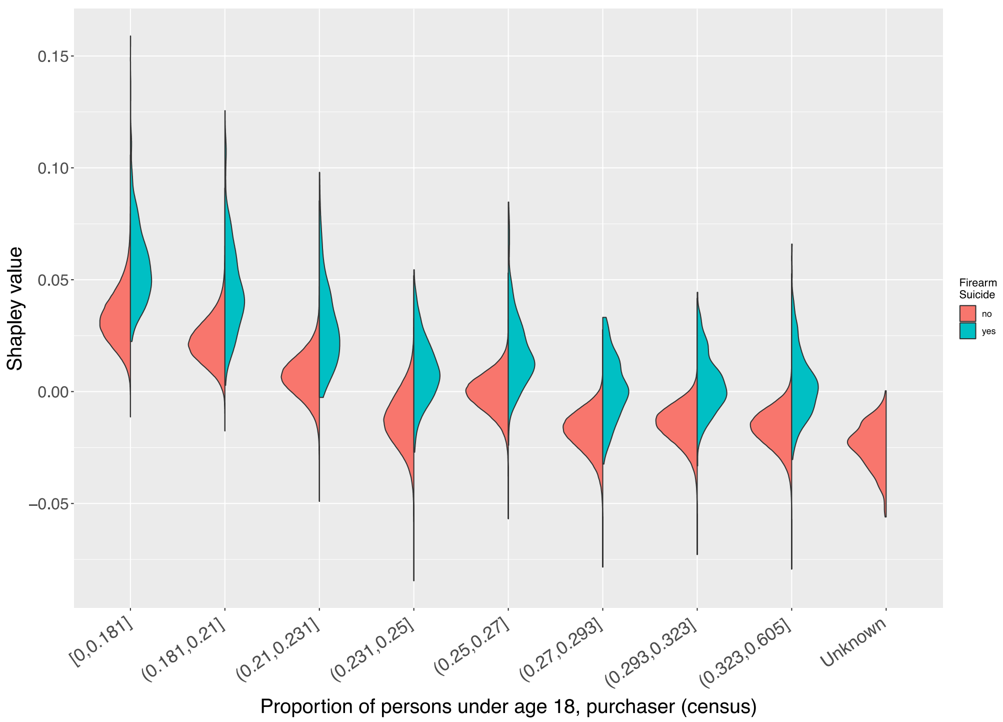

The SHAP summary plot of feature contribution (eFigure 2) is largely consistent with the Mean Decrease in Accuracy (MDA) Variable Importance (VI) reported in the manuscript. The number of previous transactions, firearm category, distance to dealer, race/ethnicity, age, and the percentage of the community population under 18 are the top contributing features. The violin plots (eFigure 3 – 8) confirm and provide further clarification on the relationships we discussed in the Results section of the manuscript. For example, among the firearm categories, the purchase of a revolver increase the probability of classification to the positive class. Having no prior transactions increases the probability of a positive class prediction as does older age at transaction.

© 2022 Laqueur HS et al. *JAMA Network Open.*

eFigure 8. Monthly Trends in Firearm Suicides

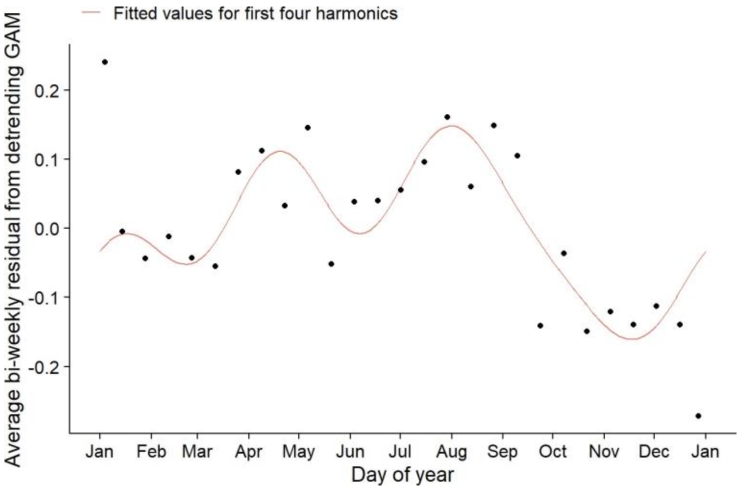

eFigure 2 shows the seasonal patterns of firearm suicide in California based on mortality data 1996 - 2016. After de-trending secular trends with a Generalized Additive Model (GAM), we modelled the Pearson residuals with cosinor terms for the first four yearly harmonics.[29] All terms are statistically significant, and their fitted values are shown plotted with the bi-weekly average.

© 2022 Laqueur HS et al. *JAMA Network Open*.

eTable. Additional Community Characteristics (Transactions Associated With Individuals Who Died by Firearm Suicide Within 1 Year)

	Firearm Suicide	Non-firearm suicide
Purchaser census tract variables		
Bachelor's degree (among 25+), mean (SD)	0.33 (0.18)	0.29 (0.17)
Proportion under 18, mean (SD)	0.23 (0.07)	0.25 (0.06)
Proportion female headed household, mean (SD)	0.22 (0.11)	0.21 (0.10)
Population per sq. kilometer, mean (SD)	2639.42 (3061.49)	2103.42 (2351.71)
Ratio male to female 15-34, mean (SD)	1.07 (0.72)	1.11 (3.14)
Unemployment rate, mean (SD)	0.08 (0.04)	0.08 (0.04)
County-level variables		
RUC Code, most rural (purchaser), No. (%)	0 (0)	66 (0.001)
RUC Code, other/unknown (purchaser), No. (%)	708 (21.60)	1489007 (29.94)
RUC Code, most urban (retailer), No. (%)	3048 (92.98)	4520020 (90.89)
RUC Code, most rural (retailer), No. (%)	2 (0.061)	6005 (0.12)
RUC Code, other/unknown (dealer), No. (%)	228 (6.96)	447088 (8.99)
FS/S (purchaser county), mean (SD)	0.522 (0.07)	0.534 (0.07)
FS/S (dealer county), mean (SD)	0.523 (0.06)	0.533 (0.06)

© 2022 Laqueur HS et al. *JAMA Network Open.*

eReferences.

1. California Penal Code 27505.
2. Fjestad SP, Fjestad SP. *Blue book of gun values.* Blue Book Publications; 2017.
3. The Census Geocoder. https://geocoding.geo.census.gov. Accessed.
4. SAS Institute Inc. SAS/STAT Software, Version 9.1. http://www.sas.com. Published 2003. Accessed.
5. Google, Inc. The Google Geocoding API.
6. GeoLytics I. Neighborhood Change Database. 2020.
7. Breiman L. Random forests. *Machine learning.* 2001;45(1):5-32.
8. Fernández-Delgado M, Cernadas E, Barro S, Amorim D. Do we need hundreds of classifiers to solve real world classification problems? *The journal of machine learning research.* 2014;15(1):3133-3181.
9. Muchlinski D, Siroky D, He J, Kocher M. Comparing random forest with logistic regression for predicting class-imbalanced civil war onset data. *Political Analysis.* 2016;24(1):87-103.
10. Wheeler AP, Steenbeek W. Mapping the risk terrain for crime using machine learning. *Journal of Quantitative Criminology.* 2021;37(2):445-480.
11. Berk R, Sherman L, Barnes G, Kurtz E, Ahlman L. Forecasting murder within a population of probationers and parolees: a high stakes application of statistical learning. *Journal of the Royal Statistical Society: Series A (Statistics in Society).* 2009;172(1):191-211.
12. Khalilia M, Chakraborty S, Popescu M. Predicting disease risks from highly imbalanced data using random forest. *BMC medical informatics and decision making.* 2011;11(1):1-13.
13. Roy A, Nikolitch K, McGinn R, Jinah S, Klement W, Kaminsky ZA. A machine learning approach predicts future risk to suicidal ideation from social media data. *NPJ digital medicine.* 2020;3(1):1-12.
14. Hastie T, Tibshirani R, Friedman JH, Friedman JH. *The elements of statistical learning: data mining, inference, and prediction.* Vol 2: Springer; 2009.
15. Fernández A, García S, Galar M, Prati RC, Krawczyk B, Herrera F. *Learning from imbalanced data sets.* Vol 10: Springer; 2018.
16. Youden WJ. Index for rating diagnostic tests. *Cancer.* 1950;3(1):32-35.
17. He H, Garcia EA. Learning from imbalanced data. *IEEE Transactions on knowledge and data engineering.* 2009;21(9):1263-1284.
18. Kuhn M, Johnson K. *Applied predictive modeling.* Vol 26: Springer; 2013.
19. Kuhn M, Wing J, Weston S, et al. caret: Classification and Regression Training. R package version 6.0-86. *Astrophysics Source Code Library: Cambridge, MA, USA.* 2020.
20. Liaw A, Wiener M. Classification and regression by randomForest. *R news.* 2002;2(3):18-22.
21. He H, Ma Y. *Imbalanced learning: foundations, algorithms, and applications.* Wiley-IEEE Press; 2013.
22. Sokolova M, Japkowicz N, Szpakowicz S. Beyond accuracy, F-score and ROC: a family of discriminant measures for performance evaluation. Paper presented at: Australasian joint conference on artificial intelligence2006.
23. Martínez-Camblor P, Pardo-Fernández JC. The Youden index in the generalized receiver operating characteristic curve context. *The international journal of biostatistics.* 2019;15(1).
24. Strobl C, Boulesteix A-L, Zeileis A, Hothorn T. Bias in random forest variable importance measures: Illustrations, sources and a solution. *BMC bioinformatics.* 2007;8(1):1-21.
25. Vigil A. *Building explainable random forest models with applications in protein functional analysis*, San Francisco State University; 2016.
26. Lundberg SM, Lee S-I. A unified approach to interpreting model predictions. *Advances in neural information processing systems.* 2017;30.
27. Greenwell B, Greenwell MB. Package 'fastshap: Fast Approximation Shapley Values.' R package. Version 0.0.7. 2021.
28. Shapley LS, Shubik M. A method for evaluating the distribution of power in a committee system. *American political science review.* 1954;48(3):787-792.
29. Barnett AG, Dobson AJ. *Analysing seasonal health data.* Vol 30: Springer; 2010.

© 2022 Laqueur HS et al. *JAMA Network Open.*
